# Classification of Skin Disease using Ensemble Data Mining Techniques

**DOI:** 10.31557/APJCP.2019.20.6.1887

**Published:** 2019

**Authors:** Anurag Kumar Verma, Saurabh Pal, Surjeet Kumar

**Affiliations:** *Research Scholor, MCA Department, VBS Purvanchal University, Jaunpur, India. *

**Keywords:** Primary health care, health information systems, skin disease, dermatology, support vector machines

## Abstract

**Objective:**

Skin diseases are a major global health problem associated with high number of people. With the rapid development of technologies and the application of various data mining techniques in recent years, the progress of dermatological predictive classification has become more and more predictive and accurate. Therefore, development of machine learning techniques, which can effectively differentiate skin disease classification, is of vast importance. The machine learning techniques applied to skin disease prediction so far, no techniques outperforms over all the others.

**Methods:**

In this research paper, we present a new method, which applies five different data mining techniques and then developed an ensemble approach that consists all the five different data mining techniques as a single unit. We use informative Dermatology data to analysis different data mining techniques to classify the skin disease and then, an ensemble machine learning method is applied.

**Results:**

The proposed ensemble method, which is based on machine learning was tested on Dermatology datasets and classify the type of skin disease in six different classes like include C1: psoriasis, C2: seborrheic dermatitis, C3: lichen planus, C4: pityriasis rosea, C5: chronic dermatitis, C6: pityriasis rubra. The results show that the dermatological prediction accuracy of the test data set is increased compared to a single classifier.

**Conclusion:**

The ensemble method used on Dermatology datasets give better performance as compared to different classifier algorithms. Ensemble method gives more accurate and effective skin disease prediction.

## Introduction

The skin is the most significant part of human body. The skin protects the body from UV radiation infections, injuries, heat and harmful radiation, and also helps in the manufacture of vitamin D. The skin plays an important role in controlling body temperature, so it is important to maintain good health and protect the body from skin diseases.

The fast development of computer technology in present decades, the use of data mining technology plays a crucial role in the analysis of skin diseases. Researchers are constantly developing various prediction methods, but the largest researchers use only a few classification algorithms instead of ensemble methods. The ensemble method uses different data mining techniques and combines them to find predictions.

Ramya and Rajeshkumar (2015) discussed the Gray-Level Co-Occurrence Matrix (GLCM) technique for finding features from segmented disease and classifying skin disease based on fuzzy classification, which is more accurate than existing ones.

Ahmed et al., (2013) discussed clusters of preprocessed data, using k-means clustering algorithms to separate related and unrelated data into skin disease. Frequent patterns were evaluated using the MAFIA algorithm. decision tree and AprioriTid algorithms are used to extract frequent patterns from clustered data sets.

Vijaya (2015) focuses on non-melanoma skin cancer and classifies types, using support vector machines (SVM) to accurately predict disease types. The chrominance and texture features are extracted pre-processed training data sets.

Chang and Chen (2009) discussed decision tree combined with neural network classification methods to construct the best predictive model of dermatology. The learning predicted and analyzed six common skin conditions. All classification techniques can predict disease fairly accurately, and the neural network model has the highest accuracy of 92.62%.

Fernando et al., (2013) discussed a disease prediction method, DOCAID, to predict malaria, typhoid fever, jaundice, tuberculosis and gastroenteritis based on patient symptoms and complaints using the naive Bayesian classifier algorithm. The authors reported an accuracy rate of 91% for predicting disease.

Theodorali et al., (2010) developed a predictive model to predict the final outcome of a seriously injured patient after an accident. The investigation includes a comparison of data mining techniques using classification, clustering, and association algorithms. Using this analysis, they obtained results in terms of sensitivity, specificity, positive predictive value, and negative predictive value, and compared results between different predictive models.

Sharma and Hota (2013) used SVM and ANN data mining techniques, to classify various types of erythema-squamous diseases. They used a confidential weighted voting scheme to combine the two technologies to achieve the highest accuracy of 99.25% in the training and 98.99% in the testing phases.

Rambhajani et al., (2015) used Bayesian classification to classify the Erythemato - Squamous disease dataset. Author used Best First Search feature selection technology technique, and they removed 20 features from the dermatology dataset collection collected by the University of California Irving repository and then used Bayesian technology to achieve 99.31% accuracy.

Bapko and Kabri (2011) in used ANN for diagnosis of different skin diseases and they achievd 90% accuracy. There are few unique features for skin cancer regions.

Yadav and Pal (2019) discussed about women thyroid prediction using data mining techniques. They used two ensemble techniques. The first ensemble technique generated by decision tree and second was generated by bagging and boosting techniques. They observed dataset for thyroid symptom and find better accuracy results.

Jaleel et al., (2012) extracted these features using a 2D wavelet transform method and then classified them using a Back Propagation Neural Network (BPNN). They classify the data set as cancer or non-cancer. 

Manjusha et al., (2014) predicting different skin diseases using the naive Bayesian algorithm. Automatic identification of circulatory disease dermatological features extracted from Local Binary Pattern from affected skin images and used for classification.

In these investigations, the main work of the differential analysis of erythematous squamous disease is Table 1.

In this research paper an attempt is done to use machine learning methods to ensemble five different data mining methods, which are Classification and Regression Trees (CART), Support Vector Machines (SVM), Decision Tree (DT), Random Forest (RF) and Gradient Boosting Decision Tree (GBDT). By merging these five data mining techniques, we construct an ensemble model to predict skin disease. Individually all five techniques are applied on the skin disease dataset. After that, a machine learning technique is designed to ensemble the results of all the five data mining methods to obtain the final result. The obtained final prediction results show that the proposed ensemble method generates more efficient use of the dataset and give more accurate result than individual data mining techniques.

## Materials and Methods

**Figure 1:**
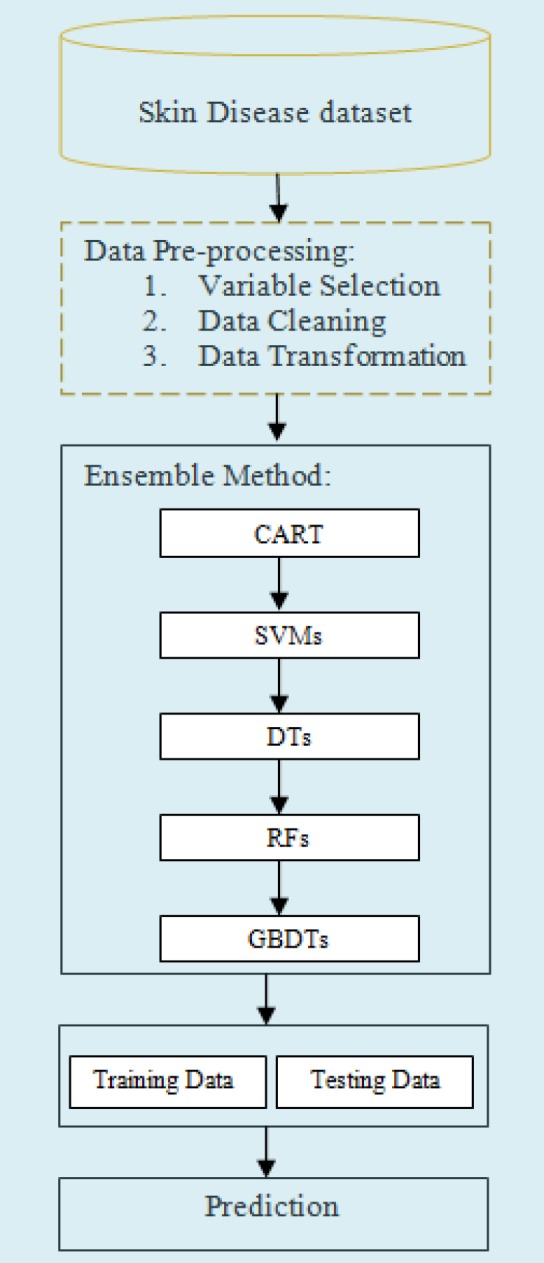
Methodological Approach for Skin Disease

Machine Learning is the technique for developing new algorithms, which provides computer the capability to learn from previously stored information’s. Fig. 1 demonstrates the whole structure of methodology used in this research paper. Figure demonstrate the different data mining methods (i) CART (ii) SVM, (iii) Decision Tree (DT), (iv) Random Forest (RF) and (v) GBDT. The approach used in this paper is completely data driven and as a number of advantages over previously used techniques. 

*Classification and Regression Trees (CART)*

The CART algorithm is based on Classification and Regression Trees. A classification tree is an algorithm where the target variable is fixed or categorical. The algorithm is then used to identify the “class” within which a target variable would most likely fall. These are examples of simple binary classifications where the categorical dependent variable can assume only one of two, mutually exclusive values. A regression tree refers to an algorithm where the target variable is and the algorithm is used to predict its value. 

*Support Vector Machine (SVM)*

Support Vector Machines is a supervised machine learning algorithm which can be used for both classification or regression challenges. However, it is mostly used in classification problems. In this algorithm, we plot each data item as a point in n-dimensional space (where n is number of features you have) with the value of each feature being the value of a particular coordinate. Then, we perform classification by finding the hyper-plane that differentiate the two classes very well. Support Vectors are simply the co-ordinates of individual observation. Support Vector Machine is a frontier which best segregates the two classes (hyper-plane/ line).

*Decision Tree (DT)*

Decision Trees are a type of Supervised Machine Learning. Decision trees are constructed via an algorithmic approach that identifies ways to split a data set based on different conditions. The goal is to create a model that predicts the value of a target variable by learning simple decision rules inferred from the data features. In decision analysis, a decision tree can be used to visually and explicitly represent decisions and decision making.

*Random Forest (RF)*

The random forest classifier can use for both classification and the regression task. Random forest classifier will handle the missing values. When we have more trees in the forest, random forest classifier won’t over fit the model. Random Forest can model the classifier for categorical values also.

*Gradient Boosting*

Gradient Boosting Machine (GBM) is a learning tool based on gradient lifting algorithm. The GBM can choose different learning algorithms as basic learners. GBDT is actually a case of GBM. Gradient Boosted Decision Trees (GBDT) is a machine learning algorithm that iteratively constructs an ensemble of weak decision tree learners through boosting.

*Dataset Analysis*

The database used in this study is taken from UCI machine repository Guvenir et al., (1998). Briefly, this dataset was formed to examine skin disease and classify type of erythemato-squamous diseases. This dataset contains 35 variables, in this dataset 34 variables are linear and 1 variable is nominal. In dermatology, erythemato-squamous disease identification and diagnosis is a difficult because all the classes contribute to the same clinical properties of scaling and erythema, with minor changes. These six classes of skin disease include C1: psoriasis, C2: seborrheic dermatitis, C3: lichen planus, C4: pityriasis rosea, C5: chronic dermatitis, C6: pityriasis rubra. Biopsy is one of the basic reatment in diagnosing these diseases. A disease may also contain the properties of another class of disease in the initial stage, which is another difficulty faced by dermatologists when performing the different class of diagnosis of these diseases. Initially patients were first examined with 12 clinical features, after which the assessment of 22 histopathological attributes was performed using skin disease samples. Histological features were identified by analyzing the samples under a microscope. If any diseases are find in the family, the family history attribute in the dataset constructed for the domain has a value of 1 (one), and if not find, the value is 0 (zero). The age of the patient is used to indicate age characteristics. All other attribute (clinical and histopathological both) were assigned a value in the range from 0 to 3 (0 = absence of features; 1, 2 = comparative intermediate values; 3 = highest value). There are six classes of erythemato-squamous disease, with 366 instances and 35 attributes in the domain. Table 2 summarizes the contents of the attributes.

*Data Preprocessing*

The methodology proposed in this research paper starts with data preprocessing. Data preprocessing step includes (i) a data driven method to select patient records and selecting important variables for analysis and (ii) The collected data from patient records are not clean and may include noise, incorrect, missing values, or inconsistent data. So we have to apply different method of data cleaning to clean such anomalies. (iii) The data are not ready for mining even after cleaning, because they are in different formats, which directly can’t be used, so data must be transform into formats suitable for mining. The transformation applied to achieve this is normalization; smoothing, aggregation, etc. are used.

*Ensembles Method*

**Figure 2:**
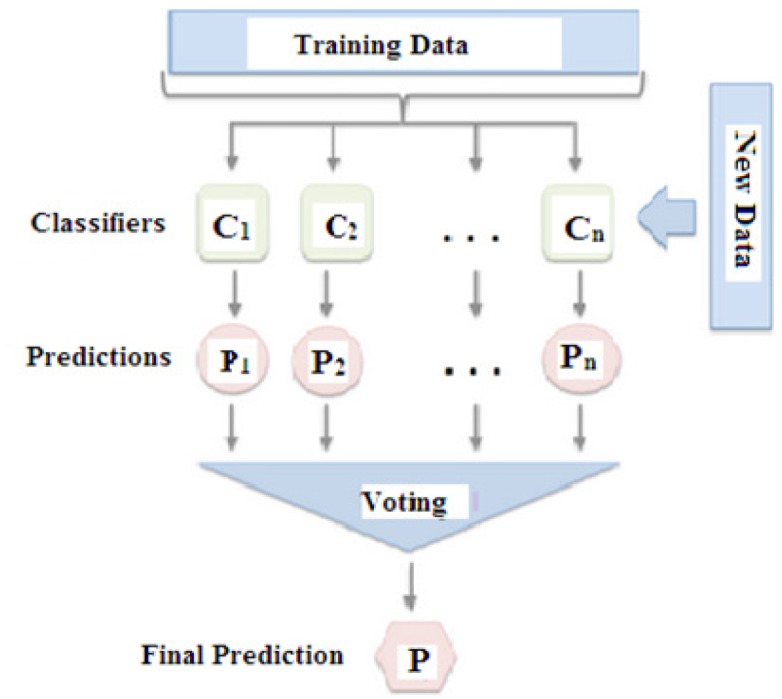
Ensemble Techniques

In this research paper ensemble method is used as a method to find the accuracy of the skin disease dataset to improve the performance of algorithms. We will evaluate five different ensemble machine learning algorithms using Gradient Boosting Decision Trees (GBDTs) See Figure 2.

## Results

**Figure 3:**
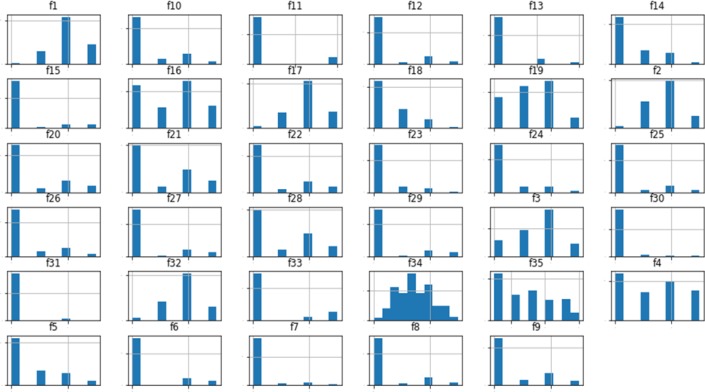
Visualization of Skin Disease Dataset

After applying the preprocessing, we try to analyze the data visually and figure out the distribution of values. Figure 3 depicts the distribution of values of erythemato-squamous disease used in our study containing 366 instances and 35 attributes.

**Figure 4:**
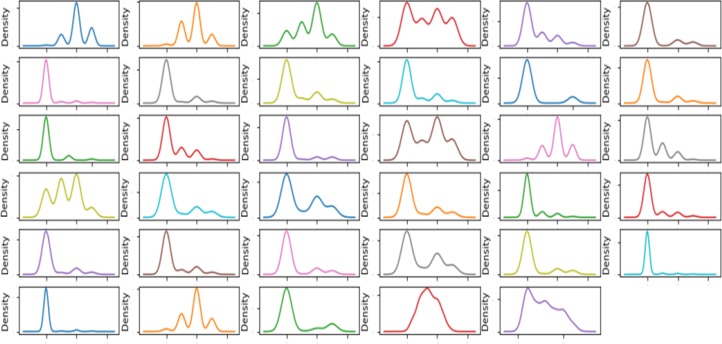
Density Map of Skin Disease Dataset

The density map is a smooth continuous version of the smoothed graph estimated from the data. The most common form of estimation is called Kernel density estimation. In this method, a continuous curve (core) is drawn at each individual data point, and then all of these curves are added together for a single smoothed density estimate. The most commonly used kernel is Gaussian (which produces a Gaussian bell curve at each data point). Density map of the attributes are illustrated in the Figure 4.

Correlation matrix is a table representing correlation coefficients between variable groups. When two variables move in the similar direction, then two variables are positively correlated. Otherwise If two variables move in opposite direction (one rising, one falling), then they are negatively correlated.

**Figure 5:**
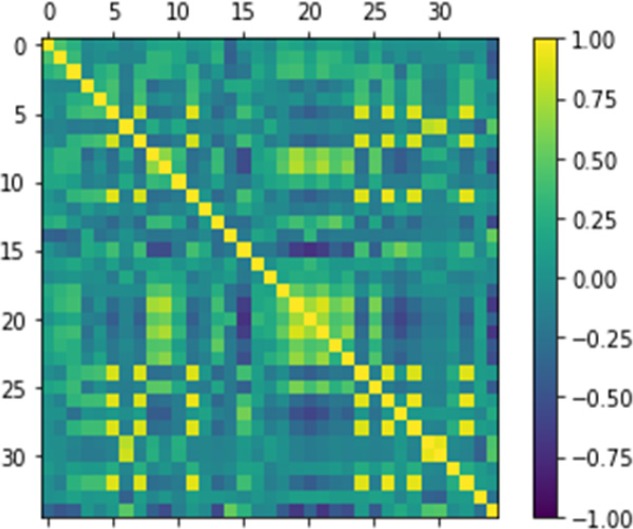
Correlation Matrix

We can calculate the correlation between each pair of attributes. This is called the correlation matrix. Then we can draw the correlation matrix and see which variables are highly correlated. This is useful because some machine learning algorithms, such as linear and logistic regression, can have poor performance if there are highly correlated input variables. The correlation matrix is shown in Figure 5.

We have used Python code to find the prediction on skin diseases dataset to calculate the accuracy and sensitivity of the five different data mining techniques initially. Python programming is chosen because the codes for different classifiers have been defined in the form of predefined modules. The value calculated by five classifiers is shown in Table 3.

**Figure 6:**
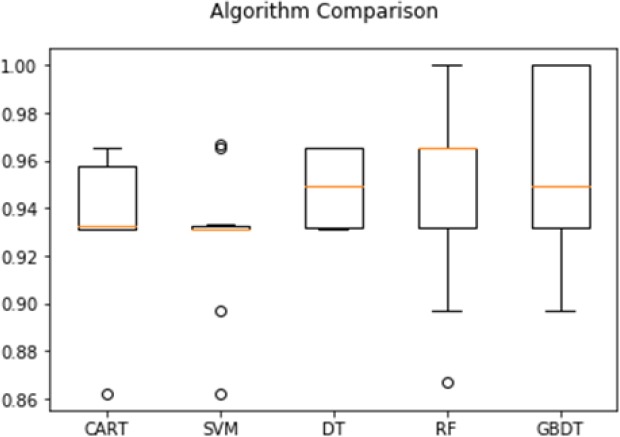
Accuracy of Different Algorithms

Another diagram that helps summarize the observed distribution is the box and the whisker. The plot draws a 25^th^ and 75^th^ percentile around the data that captures the middle 50% of the observations. Draw a line at the 50^th^ percentile (median) and draw whiskers above and below the box to summarize the general range of observations. Draw points for outliers outside the data or for outliers outside the range. The box and whisker plot of five classifier methods are shown in Figure 6.

**Figure 7:**
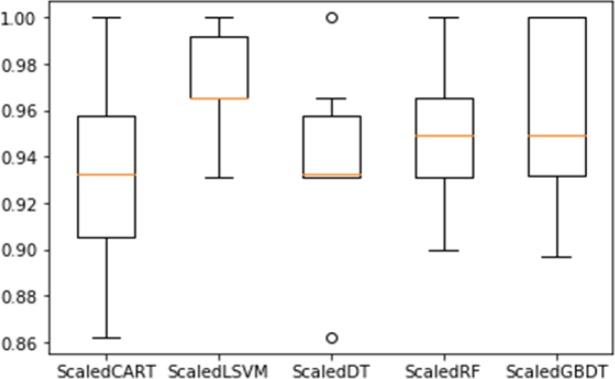
Accuracy of Different Scaled Algorithms

Variables containing discrete row values (such as AGE) are scaled to values between 0 and 10. This is done to normalize the proportional difference between each successive variable from 0 to 10, and also because the data in the selected data set for most variables varies from 0 to 10. We didn’t see the class of the normalization process because it is the target value and is not used in the process. In the case of categorical variables, we use a binary encoding process in which each categorical variable is converted into a set of binary variables so that each categorical value is associated with a binary variable. After this conversion, all nominal variables are treated as numeric variables in the {0,1} domain. After scaling all the five method are again applied on the dataset and the results obtained are shown in Table 4, and Figure 7. 

Here, we observe that accuracy of all the methods increased in comparison to without scaled data mining techniques. Now, we ensemble all the five techniques as one and perform the analysis and results are shown in Table 5.

**Table 1:** A Few Investigations which have Dealt with Skin Disease Mining

Author	Year	Method	Classification accuracy (Percentage)
Guvenir et al.	1998	VFI5	96.2
Guvenir and Emeksiz	2000	Nearest Neighbor classifier	
		Naïve Bayesian classifier	99.2
		VFI5	
Bojarczuka et al.	2001	A constrained-syntax genetic	96.64
		programmingC4.5	89.12
Ubeyli and Guler	2005	ANFIS	95.5
Nani	2006	LSVM	97.22
		RS	97.22
		B1_5	97.5
		B1_10	98.1
		B1_15	97.22
		B2_5	97.5
		B2_10	97.8
		B2_15	98.3
Polat and Gunes	2009	C4.5 and one-against-all	96.71
Ubeyli	2009	CNN	97.77
Chang and Chen	2009	decision tree	80.33
		neural network	92.62
Ubeyli and Dogdu	2010	K-mean clustering	94.22
Lekka andMikhailov	2010	Evolving fuzzy classification	97.55
Xie and Wang	2011	IFSFS and SVM	98.61
Amarathunga et al.	2015	AdaBoost	85 for Eczema
		BayesNet	95 for Impetigo
		J48, MLP (NaiveBayes)	85for Melanoma.
Parikh et al.	2015	ANN	97.17
		SVM	94.04
Parvin and Jafar	2017	Multi-SVM	97.4
		KNN	90
		Naive Bayesin	55

**Table 2:** Skin Disease Dataset

Classes	Clinical	Histopathological Attributes
C1: psoriasis	fl: erythema	f12: melanin incontinence
C2: seborrheic dermatitis	f2: scaling	f13: eosinophils in the infiltrate
C3: lichen planus	f3: definite borders	f14: PNL infiltrate
C4: pityriasis rosea	f4: itching	f15: fibrosis of the papillary dermis
C5: chronic dermatitis	f5: koebner phenomenon	f16: exocytosis
C6: pityriasis rubra	f6: polygonal papules	f17: acanthosis
	f7: follicular papules f18: hyperkeratosis	f19: parakeratosis
	f8: oral mucosal	f20: clubbing of the rete ridges involvement
	f9: knee and elbow	f21: elongation of the rete ridges
	f10: scalp involvement	f22: thinning of the suprapapillary epidermis
	f11: family history	f23: spongiform pustule
	f34: age	f24: munro microabscess
		f25: focal hypergranulosis
		f26: disappearance of the granular layer
		f27: vacuolization and damage of basal layer
		f28: spongiosis
		f29: saw-tooth appearance of rete ridges
		f30: follicular horn plug
		f31: perifollicular parakeratosis
		f32: inflammatory mononuclear infiltrate
		f33: band-like infiltrate

**Table 3:** Output of Evaluating Algorithms

Algorithms	Accuracy (Percentage)	Sensitivity ( Percentage )
CART	93.49	91.12
SVM	92.79	90.78
DT	94.87	91.13
RF	94.89	91.56
GBDT	95.9	92.38

**Table 4:** Output of Evaluating Algorithms on the Scaled Dataset

Algorithms	Accuracy ( Percentage )
ScaledCART	94.17
ScaledLSVM	96.93
ScaledDT	93.82
ScaledRF	97.27
ScaledGBDT	96.25

**Table 5:** Output of Evaluating Ensemble Method

accuracy_score	98.64%				
confusion_matrix	[[24 0 0 0 0 0]				
	[ 0 10 0 0 0 0]				
	[ 0 0 11 0 0 1]				
	[ 0 0 0 13 0 0]				
	[ 0 0 0 0 11 0]				
	[ 0 0 0 0 0 4]]				
classification_report		precision	recall	f1-score	support
	cronic dermatitis	1.00	1.00	1.00	24.00
	lichen planus	0.91	1.00	0.95	10
	pityriasis rosea	1.00	1.00	1.00	11.0
	pityriasis rubra pilaris	1.00	0.93	0.96	14.0
	psoriasis	1.00	1.00	1.00	11
	seboreic dermatitis	1.00	1.00	1.00	4.0
	avg / total	0.99	0.99	0.99	74.0

## Discussion

This research has helped to develop a collection method for predicting skin diseases. This research is the latest discovery, because to date, regulators and medical institutions have never had a comprehensive plan for developing information systems. This may be due to limited human resource capacity with expertise in formation technology and insufficient human resources for information systems.

This paper develops information system using UCI Skin disease dataset which contains 366 instances and 35 attributes. Skin dataset consists of six classes of skin disease C1: psoriasis, C2: seborrheic dermatitis, C3: lichen planus, C4: pityriasis rosea, C5: chronic dermatitis, C6: pityriasis rubra. 

Five different classification methods are chosen to perform the study (i) Classification and Regression Trees (CART) (ii) Support Vector Machines (SVMs), (iii) Decision Trees (DTs), (iv) Random Forest (RFs) and (v) Gradient Boosting Decision Trees (GBDTs). After performing these techniques we obtained the highest accuracy is 95.90 %.

we use a binary encoding process in which each categorical variable is converted into a set of binary variables so that each categorical value is associated with a binary variable. After this conversion, all the five data mining techniques are again applied on the dataset and the results obtained the highest accuracy is 97.27 %.

The performance demonstrated by the ensemble data mining techniques for skin disease prediction lies in input variable choice and classification method selection. The parameters, which are most appropriate for skin disease prediction, must be utilized as the inputs of the model. For this reason, collection of CART, SVM, DT, RF and GBDT are appropriate for classification of the Skin disease dataset in the erythemato-squamous disease identification in the ensemble test, where all five methods collectively was applied, the highest obtained accuracy is 98.64 %. 

To illustrate the success of our approach, the results obtained in this study were compared to other results given in the literature. In order to compare the efficiency of the proposed dermatological classification, we used a large number of technical studies using the same information but using different classifications techniques and then developing multi-model ensemble method. According to these studies, the same partitions of the above test data sets were followed. To illustrate this, the classification efficiency is compared to previous studies. This is shown in Table 1. Most of the same data segmentation was used as the model we presented in the study mentioned in Table 1.

In conclusion, data mining is important in healthcare organizations. Knowledge gained using data mining techniques can be used to make successful and effective decisions that improve and develop healthcare organizations. This paper describes different data mining techniques for skin disease prediction. Five machine learning techniques CART, SVM, Decision Tree (DT) , Random Forest (RF) and GBDT are used to classify the prediction of skin disease. The best accuracy find among these different techniques is 95.90% from GBDT. Then we have scaled the dataset and again applied these techniques and get higher accuracy 97.27% in case of ScaledRF. 

A multi-model ensemble method is then applied combining these five data mining technique we get the highest accuracy of 98.64%. We get the highest accuracy in the literature available on skin disease dataset. The machine learning-based multi-model collection method reduces generation errors and obtains more information by using the first-stage prediction as a feature rather than a separate training. In addition, by using machine learning, the complex relationships between classifiers are automatically learned, enabling the collection method for better predictions.

## Conflicts of interest

The authors made no conflict of interest.
